# Arterial Stiffening in Western Diet-Fed Mice Is Associated with Increased Vascular Elastin, Transforming Growth Factor-β, and Plasma Neuraminidase

**DOI:** 10.3389/fphys.2016.00285

**Published:** 2016-07-07

**Authors:** Christopher A. Foote, Jorge A. Castorena-Gonzalez, Francisco I. Ramirez-Perez, Guanghong Jia, Michael A. Hill, Constantino C. Reyes-Aldasoro, James R. Sowers, Luis A. Martinez-Lemus

**Affiliations:** ^1^Dalton Cardiovascular Research Center, University of MissouriColumbia, MO, USA; ^2^Department of Biological Engineering, University of MissouriColumbia, MO, USA; ^3^Diabetes and Cardiovascular Research Center, University of MissouriColumbia, MO, USA; ^4^Harry S. Truman Memorial Veterans HospitalColumbia, MO, USA; ^5^Department of Medical Pharmacology and Physiology, University of MissouriColumbia, MO, USA; ^6^School of Engineering and Mathematical Sciences, City University LondonLondon, UK

**Keywords:** vascular compliance, neuraminidase, TGF-β, vascular remodeling, overnutrition

## Abstract

Consumption of excess fat and carbohydrate (Western diet, WD) is associated with alterations in the structural characteristics of blood vessels. This vascular remodeling contributes to the development of cardiovascular disease, particularly as it affects conduit and resistance arteries. Vascular remodeling is often associated with changes in the elastin-rich internal elastic lamina (IEL) and the activation of transforming growth factor (TGF)-β. In addition, obesity and type II diabetes have been associated with increased serum neuraminidase, an enzyme known to increase TGF-β cellular output. Therefore, we hypothesized that WD-feeding would induce structural modifications to the IEL of mesenteric resistance arteries in mice, and that these changes would be associated with increased levels of circulating neuraminidase and the up-regulation of elastin and TGF-β in the arterial wall. To test this hypothesis, a WD, high in fat and sugar, was used to induce obesity in mice, and the effect of this diet on the structure of mesenteric resistance arteries was investigated. 4-week old, Post-weaning mice were fed either a normal diet (ND) or WD for 16 weeks. Mechanically, arteries from WD-fed mice were stiffer and less distensible, with marginally increased wall stress for a given strain, and a significantly increased Young's modulus of elasticity. Structurally, the wall cross-sectional area and the number of fenestrae found in the internal elastic lamina (IEL) of mesenteric arteries from mice fed a WD were significantly smaller than those of arteries from the ND-fed mice. There was also a significant increase in the volume of elastin, but not collagen in arteries from the WD cohort. Plasma levels of neuraminidase and the amount of TGF-β in mesenteric arteries were elevated in mice fed a WD, while *ex vivo*, cultured vascular smooth muscle cells exposed to neuraminidase secreted greater amounts of tropoelastin and TGF-β than those exposed to vehicle. These data suggest that consumption of a diet high in fat and sugar causes stiffening of the vascular wall in resistance arteries through a process that may involve increased neuraminidase and TGF-β activity, elevated production of elastin, and a reduction in the size and number of fenestrae in the arterial IEL.

## Introduction

Consumption of a Western Diet (WD), high in fat and sugar, has led to a dramatic increase in the prevalence of obesity [body mass index (BMI) ≥ 30 kg/m^2^] over the last decades in the U.S, and worldwide (Parikh et al., 2007; de Onis et al., 2010). Obesity is associated with impaired cardiovascular function and is a risk factor for a range of pathologies including hypertension, atherosclerosis, and Type II diabetes (Mcgill et al., 2002; Aneja et al., 2004; Guilherme et al., 2008; Stolarczyk et al., 2013). Consumption of excess nutrients and associated overweight/obesity is also linked with increased arterial stiffness. Using aortic pulse wave velocity (PWV) as a measurement of arterial stiffness, abdominal body fat was positively correlated with a faster pulse wave, indicative of stiffer arteries (Sutton-Tyrrell et al., 2001). Additional studies have also found a positive correlation between BMI and aortic PWV, suggesting there is an association between obesity and arterial stiffening (Toto-Moukouo et al., 1986; Tounian et al., 2001; Wildman et al., 2003; Majane et al., 2009). Moreover, we recently reported that mice fed a WD for 16 weeks have increased aortic and femoral PWVs (Bender et al., 2015; DeMarco et al., 2015).

Arterial stiffening is characterized by a reduction in the ability of an artery to passively expand and contract in response to luminal pressure changes. This stiffening causes a decreased level of distensibility at a given pressure that has important pathological implications, as cardiovascular disease (CVD) is associated with increased vessel stiffness (van Popele et al., 2001; Duprez and Cohn, 2007; Payne et al., 2010; Wagenseil and Mecham, 2012). Arterial stiffening is also prevalent in type II diabetes, where the subsequent development of CVD is the main cause of death (Huxley et al., 2006; Stehouwer et al., 2008). The mechanism(s) leading to arterial stiffness have not been fully elucidated, including whether vascular stiffening occurs equally in all vascular beds and at all levels of the vascular tree. We recently reported that in a mouse model of WD-induced obesity (a primary cause of type II diabetes; Bender et al., 2015), femoral arteries undergo significant structural changes that increase their stiffness. However, these changes were not observed in the coronary arteries, which suggest that vessels of the same caliber located in different vascular beds are not equally affected by consumption of a WD. Here, we aimed to define the effects of WD-feeding on the functional and structural characteristics of mesenteric resistance arteries to determine if their responses were similar to those of femoral or coronary arteries.

One of the mechanisms proposed to initiate arterial stiffening is a change in the structural organization of the extracellular matrix (ECM) within the arterial wall. Elastin and collagen fibers are the main structural ECM components of the vascular wall. Elastin in particular is the most abundant fibrous protein in vascular laminas. It generally allows the vessel to expand and return to its original diameter in response to pressure changes. In the muscular and resistance arteries from a number of vascular beds in rodents, the internal elastic lamina (IEL) is an intact sheet perforated with holes, termed fenestrae. These openings provide portals for communication between the endothelium and vascular smooth muscle cells (VSMCs) via direct cell-cell membrane contacts (Ledoux et al., 2008) or through the diffusion of vasoactive agents. It was demonstrated that the size and number of these fenestrae are reduced in inwardly remodeled vessels, that is vessels with a reduced passive luminal diameter due to structural changes, from angiotensin-II treated rodents, and this reduction coincided with an increase in mesenteric arterial stiffness and the development of hypertension in rats (Briones et al., 2009). In a pig model of atherosclerosis, the number of fenestrae was also decreased in atherosclerotic coronary arteries (Kwon et al., 1998). Moreover, in our model of diet-induced obesity, male mice fed a WD for 16 weeks had stiffer femoral arteries in which the number and size of fenestrae were reduced. Coronary arteries from the same animals had no changes in their level of IEL fenestration (Bender et al., 2015). Therefore, an additional goal of the present study was to determine whether fenestrae in the IEL of mesenteric resistance arteries change in response to consumption of a WD in a fashion similar to femoral arteries.

The observed changes in fenestration in hypertension and in response to diet-induced obesity suggest that dynamic changes of the IEL could be an early structural modification in the process that leads to the development of obesity-related cardiovascular disease. A number of studies suggest that obesity is associated with increased elastase activity leading to elevated plasma levels of elastin-derived peptides that in turn bind to their receptor and increase neuraminidase activity (Bizbiz and Robert, 1996; Talukdar et al., 2012; Blaise et al., 2013). Neuraminidase has been shown to promote elastogenesis in both human fibroblasts and human aortic smooth muscle cell cultures (Hinek et al., 2006, 2008). Neuraminidase has also been shown to increase the production and activity of transforming growth factor (TGF)-β (Carlson et al., 2010), an inflammatory cytokine that upregulates elastogenesis both transcriptionally and Post-translationally (for review, see Sproul and Argraves, 2013). In addition, it has been reported that neuraminidase is elevated in the serum and urine of human type II diabetic patients (Roozbeh et al., 2011). These reports led us to hypothesize that the structural modifications to the IEL that occur in mice fed a WD (which develop insulin resistance), may be associated with increased levels of circulating neuraminidase, leading to increased TGF-β activity and the up-regulation of elastogenesis in the vascular wall. We tested this hypothesis in mesenteric resistance arteries of male mice fed a WD for 16 weeks.

## Materials and methods

### Animals

All animal procedures were approved by Animal Use and Care Committees at the University of Missouri-Columbia and Harry S. Truman Veterans' Memorial Hospital, and performed in accordance with National Institutes of Health guidelines. C57BL6/J males were obtained from The Jackson Laboratory. Groups of 4-week-old male mice were fed a WD (TestDiet 5APC) consisting of high fat (46%) and high carbohydrate as sucrose (17.5%) and high-fructose corn syrup (17.5%) for 16 weeks (Nistala et al., 2014). A parallel group of age-matched male controls were fed a normal diet (ND), i.e., regular mouse chow, for the same period of time (TestDiet 5APD). Both cohorts were provided water *ad libitum* while housed in pairs under a 12 h/day illumination regimen.

### Assessment of insulin resistance

Blood glucose and insulin levels were determined as previously described (Zhou et al., 2010). Briefly, venous blood was drawn from fasting mice and assessed for glucose concentrations, using a G-6-PDH assay, and insulin levels, with a murine specific ELISA assay. Insulin resistance was assessed using the homeostasis model assessment (HOMA-IR; Matthews et al., 1985). Indeed, HOMA-IR was calculated using the following formula: HOMA-IR = fasting glucose (mg/dL) × fasting insulin (μU/ml)/405. HOMA-IR has been shown to be strongly correlated with the insulin sensitivity index derived from the standard euglycemic hyperinsulinemic clamp method (Emoto et al., 1999). In the HOMA-IR model, insulin levels are expressed in international units (IU). Within the literature, there is a discrepancy on the concentration of insulin that equals 1 μU of activity (Heinemann, 2010). We used the potency factor (28,698 U/g) provided by the manufacturer of Novolin R (Novo-Nordisk), thus the conversion from mass units to units of activity was 6.00 ρmol/l = 1 μU/ml.

### Vessel isolation and vascular functional assessments

Mice were anesthetized by means of isoflurane inhalation. After confirmation that spinal reflexes were lost, the mesenteric vasculature was excised and placed in a cold (~4°C) physiological saline solution (PSS) containing: 145.0 NaCl, 4.7 KCl, 2.0 CaCl_2_, 1.0 MgSO_4_, 1.2 NaH_2_PO_4_, 0.02 EDTA, 2.0 Pyruvic Acid, 5.0 Glucose and 3.0 MOPS (all concentrations are given in mM) with a final pH of 7.4. First order (1A) feed mesenteric arteries were isolated, cannulated and pressurized for experimentation as previously described (Martinez-Lemus, 2008). Briefly, arteriolar segments of ~3 mm in length were cannulated onto glass micropipettes within an observation chamber (Living Systems Instrumentation, Burlington, Vermont) filled with PSS. The arteries were pressurized without flow to 70 mmHg using a Pressure Servo System (Living Systems Instrumentation Burlington, Vermont) and PSS containing 0.15 mM bovine serum albumin. The observation chamber with the cannulated vessel was transferred to an inverted microscope equipped with a video display and video caliper system (Living Systems Instrumentation Burlington, Vermont) to record measurements of wall thickness and luminal diameter. All experiments were performed at 37°C. After warming, vessels were exposed to PSS containing 80 mM KCl equimolarly substituted for NaCl to induce depolarization and vasoconstriction and test viability. Following washout and equilibration, vessels were exposed to increasing concentrations of phenylephrine to test for adrenergic vasoconstriction responses. Subsequently, responses to increasing concentrations of insulin and sodium nitroprusside (SNP) were performed after vessels were Pre-constricted with 10^−6^ M phenylephrine to test endothelium-dependent and -independent vasodilation. Responses are reported as percent of maximal passive diameter or as percent of phenylephrine Pre-constriction. Maximal passive diameter was obtained at the end of each experiment by exposing vessels to Ca^+2^-free PSS in the presence of 2 mM EGTA and 10^−4^ M adenosine.

### Determination of arterial structural and mechanical characteristics

To study the structural and mechanical characteristics of the arteriolar wall, pressure-diameter curves were obtained under passive conditions (Ca^2+^-free PSS) at the end of each experimental protocol. Changes in intraluminal pressure were performed in steps covering a range between 5 and 120 mmHg. Internal diameter and wall (left and right) thicknesses were recorded at each pressure. This information was subsequently used to determine vascular remodeling, circumferential strain and stress, compliance, distensibilty, and the incremental modulus of elasticity curves for each group of vessels. Vascular remodeling was assessed as a change, either inward or outward, in the passive internal diameter of a vessel (Mulvany, 1999). Circumferential strain was calculated with the following equation: ϵ = (*D*_*P*_−*D*_5mmHg_)∕*D*_5mmHg_. Circumferential stress was calculated by using the following formula, where *P* is the intraluminal pressure, and τ is wall thickness: σ = (*P*·*D*_*P*_)∕2τ_*P*_. Cross-sectional compliance was calculated as the change in the CSA of the wall relative to an increment in intraluminal pressure: *C* = Δ*A*∕Δ*P* (Souza-Smith et al., 2011; Bender et al., 2015; Pennington et al., 2016). Cross-sectional distensibility was calculated using the following equation: *D* = Δ*A*∕(Δ*P*·*A*). The incremental modulus of elasticity was determined using the equation: *E*_*inc*_ = Δσ∕Δϵ (Souza-Smith et al., 2011). Vessels were subsequently fixed in 4% paraformaldehyde, while pressurized at 70 mmHg for 1 h in preparation for imaging with a confocal/multiphoton microscope. Vessels were rinsed twice in phosphate buffered saline (PBS) and once in 0.1 M Glycine for 5 min each time. After rinsing their lumen with 1 mL PBS, vessels were permeabilized via incubation in TritonX100 at 0.5% for 20 min. Vessels were then washed twice in PBS and incubated for 1 h in 0.5 μg/mL 4′,6-diamidino-2-phenylindole (DAPI), 0.2 μM Alexa Fluor 633 Hydrazide (Molecular Probes) and 0.02 μM Alexa Fluor 546 Phalloidin (Molecular Probes) in PBS. After incubation, vessels were washed 3 times in PBS and imaged using a Leica SP5 confocal/multiphoton microscope with a 63x/1.2 numerical aperture water objective. Alexa Fluor 633, which selectively labels elastin (Clifford et al., 2011; Shen et al., 2012), was excited with a HeNe laser at 633 nm. Alexa Fluor 546 Phalloidin, to image fibrillar (F)-actin components, was excited with a HeNe laser at 543 nm. DAPI, to image nuclei, was excited with a multi-photon laser at 720 nm. Collagen was imaged via second-harmonic image generation using a multi-photon laser at 850 nm. Images were processed and all channels were quantified to determine the total volume occupied by VSMC nuclei, elastin, actin (within the media), and collagen with an in-house Matlab algorithm as previously described (Bender et al., 2015).

### Determination of neuraminidase in plasma

A blood sample from each mouse was obtained at the time of euthanasia, and plasma separated by centrifugation for further analysis. Neuraminidase activity was determined in plasma using an Amplex Red Neuraminidase Assay Kit (A22178, Molecular Probes). Briefly, 5 μl of plasma (performed in duplicate) were incubated for 1 h at 37°C in Amplex Red reaction mixture and end-point fluorescence was read in a Biotek Synergy HT microplate reader using excitation at 530 nm and collection at 590 nm. End-point fluorescence was averaged for each sample, and mean values determined for mice in the ND and WD cohorts.

### Quantification of transforming growth factor β (TGF-β) in mesenteric arteries

We have previously reported increased TGF-**β** levels in femoral arteries from mice fed a WD compared to ND-fed mice (Bender et al., 2015). Since TGF-**β** is a well-characterized initiator of elastogenesis, and we were interested in identifying potential mechanism(s) that mediate elastogenesis in the resistance vasculature, we assessed TGF-**β** levels in mesenteric arteries from ND- and WD-fed mice. Vessels were fixed in 4% paraformaldehyde, while pressurized at 70 mmHg for 1 h. Vessels were rinsed twice in phosphate buffered saline (PBS) and once in 0.1 M Glycine for 5 min each time. After rinsing their lumen with 1 mL PBS, vessels were permeabilized via incubation in TritonX100 at 0.5% for 20 min. Vessels were then washed thrice in PBS, followed by a 30-min blocking in Antibody Wash Buffer (150 mM NaCl, 17 mM NaCitrate, 0.05% TritonX100, and 1% Bovine Serum Albumin). Vessels were incubated overnight in Antibody Wash Buffer containing 1/500 dilution rabbit TGF-β antibody (ab66043, Abcam), washed 5x in Antibody Wash Buffer and incubated for 1 h in Antibody Wash Buffer containing 1/1000 dilution of an AlexaFluor 488 conjugated secondary (A11034, ThermoFisher) and DAPI at a 0.5 μg/ml final concentration. Vessels were washed 5 times in Antibody Wash Buffer, rinsed 3 times in PBS and imaged using a Leica SPE confocal microscope with a 63x/1.2 numerical aperture water objective. Imaris software v8.1 was used to quantify the mean TGF-β associated fluorescent intensity/voxel within a region of interest (50 × 50 × 15 μM) encompassing the tunica intima and medial layers of the vessel wall.

### Quantification of tropoelastin and TGF-β production in cell culture

*Rat VSMCs, passage 3*, were grown to confluence in DMEM + 10% fetal bovine serum (FBS) in 60 mm dishes. Cell cultures were grown an additional 6 days in either DMEM + 1% FBS (control) or DMEM + 1% FBS supplemented with 50 mU neuraminidase (N2876, Sigma). The conditioned media was collected on day 3 and day 6, pooled, lyophilized in the presence of protease inhibitors (78430, ThermoFisher) and assayed for protein content using a BCA Protein Assay Kit (23227, Pierce). For each sample, 25 μg total protein for tropoelastin detection (37.5 μg for TGF-β) was mixed with 2x sodium dodecyl sulfate sample buffer + β-mercaptoethanol, loaded and separated on 10% w/v polyacrylamide gels for tropoelastin (7.5% gels for TGF-β) transferred to polyvinylidene difluoride membranes and probed with either a 1/500 dilution of α-elastin antibody (RA75, Elastin Products Co.) or rabbit TGF-β antibody (ab66043, Abcam). A horseradish peroxidase (HRP) conjugated secondary antibody (HAF109, R&D Systems) was used at 1/1000 dilution for chemiluminescent detection of tropoelastin, revealing three major bands, corresponding to the 72 kDa full length tropoelastin protein and two smaller tropoelastin degradation products both of which were larger than 50 kDa. Band intensity was quantified for all three bands using Bio-Rad Image Lab Software v5.1. The mean intensity of the sum of all three bands was compared between cells exposed to vehicle vs. those exposed to 50 mU neuraminidase, and normalized to control levels. For TGF-β, a 1/3000 dilution of rabbit HRP secondary (170-50456, Bio-Rad) was used for detection, and band intensity was quantified using Bio-Rad Image Lab Software v5.1.

### Data analyses

Data are expressed as means ± SEM. The number of experiments represents the number of animals in each experimental group. Statistical analyses included *T*-test, or ANOVA followed by Bonferroni *post-hoc* for multiple comparisons where appropriate. A value of *P* ≤ 0.05 was considered significant. All statistical analyses were performed using GraphPad Prism software.

## Results

### WD-feeding induced significant weight gain, hyperinsulinemia, and insulin resistance

**Figure 1:**
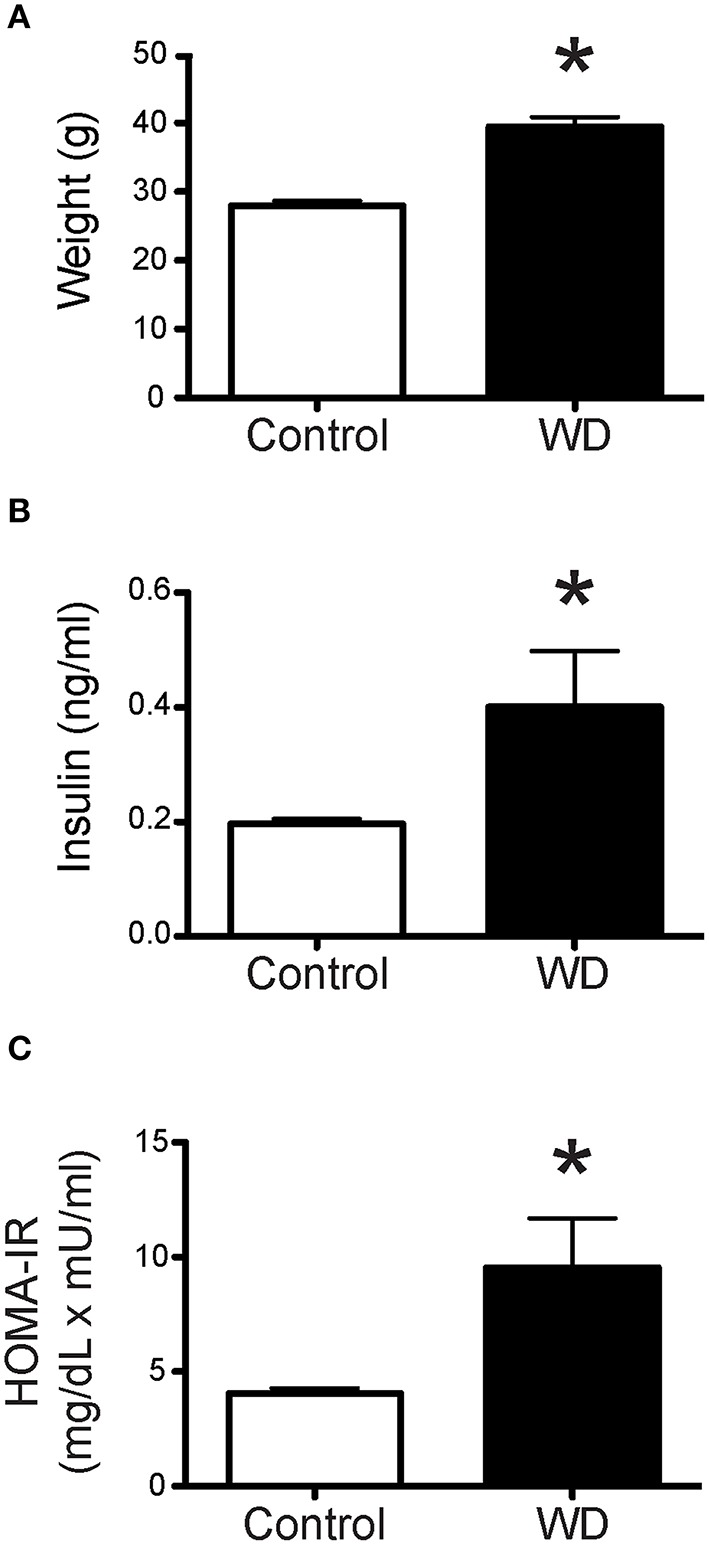
**Mice fed a WD were significantly heavier than ND-fed mice and had insulin resistance. (A)** Mean body weights of control ND-fed mice (*n* = 9) and WD-fed mice (*n* = 10). Mean body weight was greater in WD-fed mice, ^*^P < 0.05 vs. Control. **(B)** Plasma concentration of insulin from mice following 4 h of fasting, was significantly elevated in mice fed a WD (*n* = 10) vs. control mice fed a ND (*n* = 9), ^*^P < 0.05. **(C)** HOMA-IR (fasting glucose mg/dL × fasting insulin uU/ml/405) index was significantly higher for WD-fed mice (*n* = 10) vs. control mice fed a ND (*n* = 9), ^*^P < 0.05. Data are means ± SEM.

Feeding mice a WD high in fat and sugar for 16 weeks after weaning resulted in substantial weight gain compared to mice fed a ND. The average body weight of 20 week-old WD-fed mice was greater than that of mice fed a ND (*P* < 0.05, Figure 1A). In addition, there was a significant increase in fasting plasma insulin levels in the WD cohort, which in turn resulted in insulin resistance as assessed by HOMA-IR (Figures 1B,C).

### WD-feeding did not affect vascular responses to phenylephrine, K^+^, insulin, or SNP

**Figure 2:**
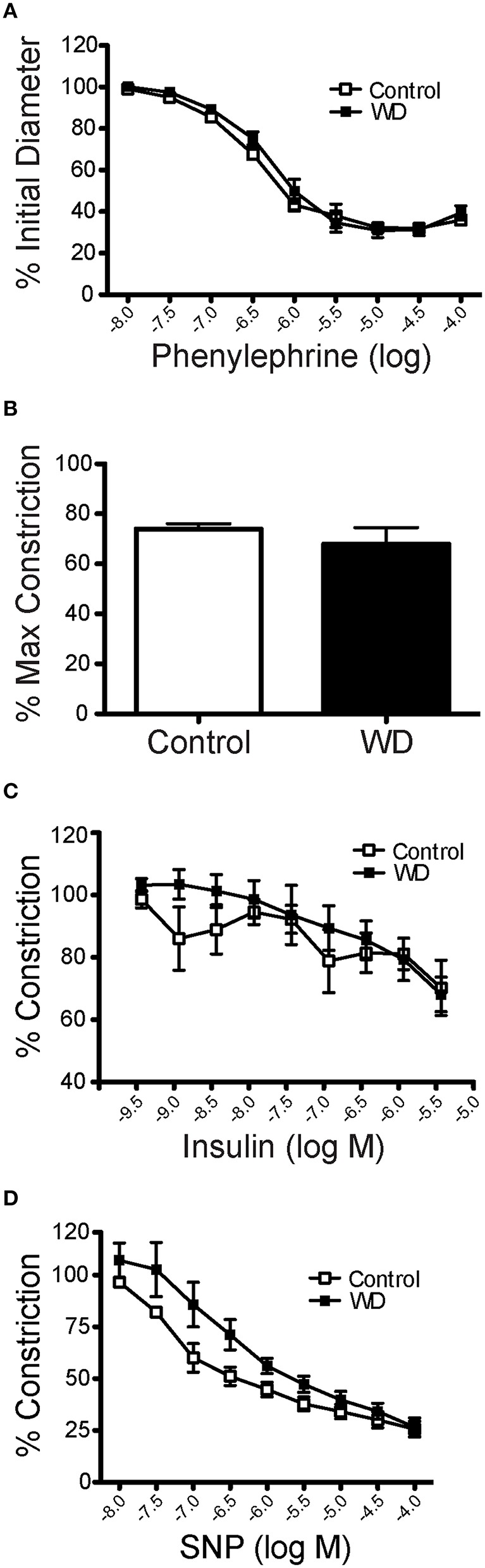
**Mesenteric artery vasomotor responses were not affected by WD consumption. (A)** Changes in internal diameter of mesenteric arteries from ND- (Control) and WD-fed mice in response to increasing concentration of phenylephrine. Vascular diameters are expressed as a percentage of the initial vessel diameter with tone. **(B)** Percent maximal constriction of mesenteric arteries from ND- (Control) and WD-fed mice exposed to 80 mM KCl. **(C)** Changes in internal diameter of mesenteric arteries from ND- (Control) and WD-fed mice in response to increasing concentration of insulin. Vascular diameters are expressed as percentage of the pre-constriction induced by 10^−6^ M phenylephrine. **(D)** Changes in internal diameter of mesenteric arteries from ND- (Control) and WD-fed mice in response to increasing concentration of SNP. Vascular diameters are expressed as percentage of the Pre-constriction induced by 10^−6^ M phenylephrine. Data are expressed as means ± SEM of *n* = 9 ND- (Control) and *n* = 10 WD-fed mice.

The overall characteristics of mesenteric arteries from ND- and WD-fed mice including their main responses to vasoactive agonists are included in Table 1. Mesenteric resistance arteries from mice fed a WD or ND had similar vasoconstriction responses when exposed to increasing concentrations of phenylephrine (Figure 2A). Consequently, there were no significant differences in maximum constriction or the half maximal effective concentration (EC50) for phenylephrine between arteries from the WD- or ND-fed mice. Maximal vasoconstriction responses to 80 mM K^+^ also did not differ between the groups (Figure 2B). Because mice fed a WD were insulin resistant, we aimed to determine whether arteries from WD-fed mice had impaired vasodilatory responses to insulin. There were no significant differences in the insulin-induced dilations between arteries from WD- and ND-fed mice (Figure 2C). No significant differences were observed between the WD- and ND-fed groups either for the endothelium-independent vasodilation induced by SNP (Figure 2D).

**Table 1:** **Characteristics of mesenteric arteries from ND (Control) and WD fed mice**.

	**Control (*n* = 9)**	**WD**
Maximal diameter, μm	217.44 ± 11.98	214.88 ± 6.80 (*n* = 10)
Tone diameter, μm	194.20 ± 14.46	211.88 ± 7.59 (*n* = 10)
Constriction KCl, % max	26.06 ± 2.12	32.04 ± 6.59 (*n* = 10)
Constriction Phe, % max	28.48 ± 2.00	34.75 ± 3.25 (*n* = 10)
**INSULIN**		
Pre-constriction, % max	41.81 ± 3.99	51.26 ± 5.49 (*n* = 9)
Vasodilation, % max	58.52 ± 5.73	66.04 ± 2.98 (*n* = 9)
**SNP**		
Pre-constriction, % max	47.67 ± 3.21	54.43 ± 2.96 (*n* = 8)
Vasodilation, % max	86.04 ± 2.58	86.00 ± 2.26 (*n* = 8)

*Values are mean ± SEM; Maximal diameter measured in Ca^2+^-free PSS; KCl concentration = 80 mM; Phenylephrine (Phe) concentration = 10^-4^ M; % max = percentage maximum passive diameter; for Insulin and SNP assessment, vessels were Pre-constricted with 10^-6^ M Phe, vaso dilation data are for 10^−5^ M insulin and 10^-4^ M SNP, respectively*.

### WD-feeding changed the mechanical and structural characteristics of mesenteric resistance arteries

**Figure 3:**
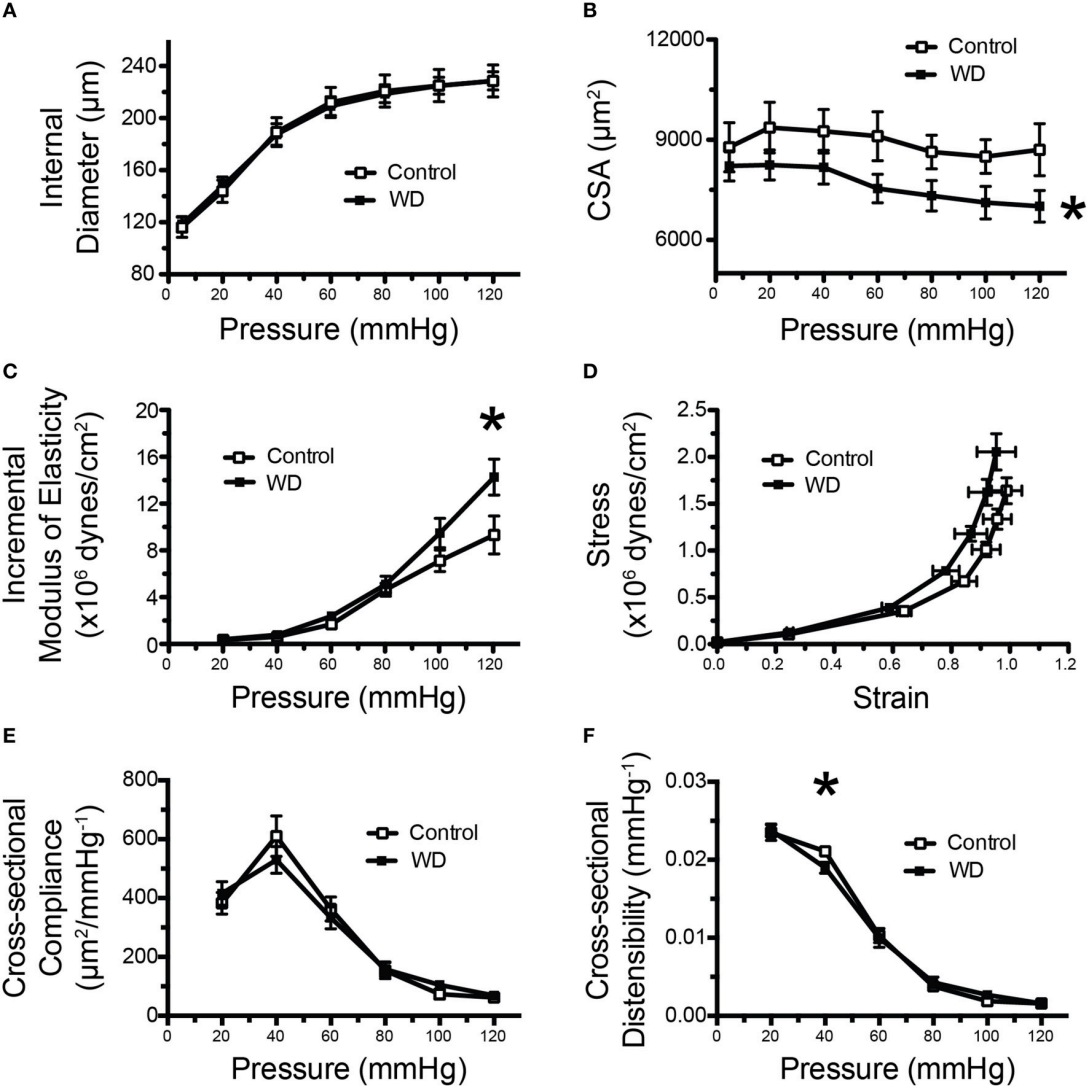
**Mesenteric arteries from WD-fed mice were stiffer than those from ND-fed mice. (A)** Pressure vs. inside wall diameter curves of mesenteric arteries from ND- (Control) and WD-fed mice. **(B)** Vascular wall cross-sectional area (CSA) of mesenteric arteries from ND- (Control) and WD-fed mice. **(C)** Incremental modulus of elasticity (stiffness) of mesenteric arteries from ND- (Control) and WD-fed mice. **(D)** Strain-stress curves of mesenteric arteries from ND- (Control) and WD-fed mice. **(E)** Cross-sectional compliance of mesenteric arteries from ND- (Control) and WD-fed mice. **(F)** Cross-sectional distensibility of mesenteric arteries from ND- (Control) and WD-fed mice. All measurements were made under passive conditions (Ca^2+^-free PSS + 10^−4^ M adenosine + 2 mM EGTA). Data are expressed as means ± SEM of *n* = 10 WD-fed and *n* = 9 ND-fed (Control) mice. ^*^P < 0.05 vs. Control.

Analyses of the passive pressure-diameter curves in mesenteric arteries indicated that consumption of a WD did not induce changes in passive luminal diameters at any pressure (Figure 3A). However, there was a non-significant tendency (*P* = 0.07) for mesenteric arteries from WD-fed mice to have a reduced wall thicknesses and a significant (*P* < 0.05) reduction in wall cross-sectional areas (CSA) compared to those of ND-fed mice (Figure 3B). We calculated both geometric-dependent and -independent properties of the arteries. The parameters independent of geometry provide information about changes to the wall material. We found a significantly greater (*P* < 0.05) incremental modulus of elasticity and a marginally (*P* = 0.07) increased wall stress as a function of strain in arteries from WD- vs. those of ND-fed mice (Figures 3C,D). For geometrically dependent parameters, cross-sectional compliance was not significantly different between the WD and ND-fed mice (Figure 3E), though, distensibility, was significantly reduced at 40 mmHg for the WD cohort (Figure 3F), indicative of increased arterial stiffness.

### WD-feeding increased mesenteric artery elastin content and changed the structural characteristics of their internal elastic laminas

**Figure 4:**
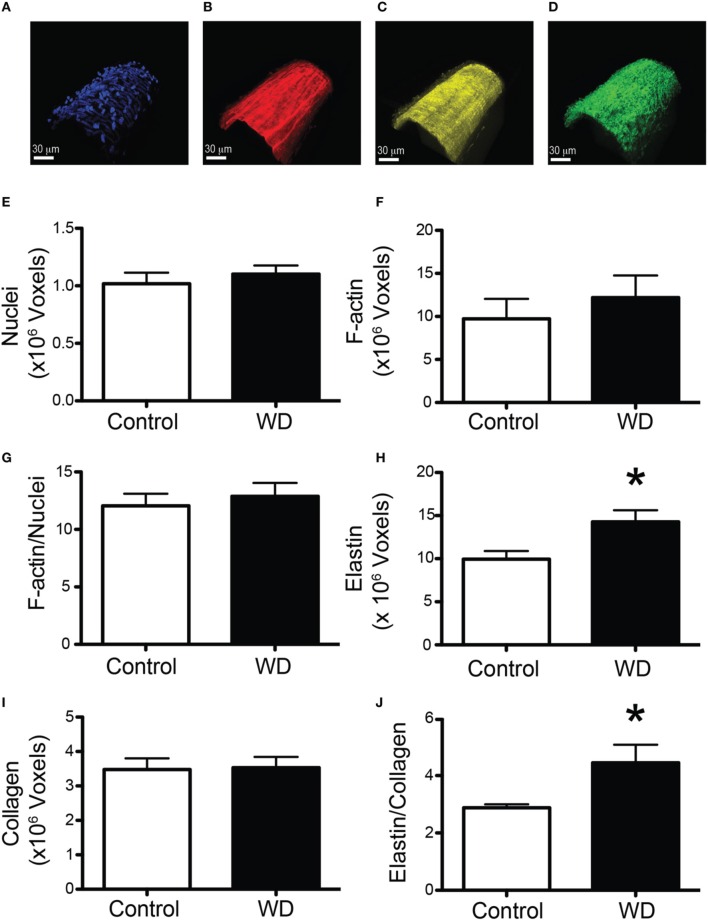
**WD-feeding increased the elastin content of mesenteric arteries. (A–D)** Representative images of a vessel stained with DAPI to image nuclei **(A)**, Alexa633 to image elastin **(B)**, Phalloidin to image actin **(C)**, and subjected to second harmonics to image collagen **(D)**, scale bar = 30 μm. **(E)** Comparison of mean number of voxels containing DAPI fluorescence in the medial layer of mesenteric arteries from ND- (Control) and WD-fed mice. **(F)** Comparison of mean number of voxels containing phalloidin fluorescence in the medial layer of mesenteric arteries from ND- (Control) and WD-fed mice. **(G)** Comparison of the ratio of the number of voxels containing phalloidin over those containing DAPI fluorescence in the medial layer of mesenteric arteries from ND- (Control) and WD-fed mice. **(H)** Comparison of mean number of voxels containing Alexa633 fluorescence in the wall of mesenteric arteries from ND- (Control) and WD-fed mice. **(I)** Comparison number of voxels containing second harmonic detected collagen in the wall of mesenteric arteries from ND- (Control) and WD-fed mice. **(J)** Comparison of the ratio of the number of voxels containing Alexa633 fluorescence over those containing second harmonic detected collagen in the wall of mesenteric arteries from ND- (Control) and WD-fed mice. In **(E–J)**, data are expressed as means ± SEM of *n* = 9 ND- (Control) and *n* = 10 WD-fed mice. ^*^P < 0.05 vs. Control.

**Figure 5:**
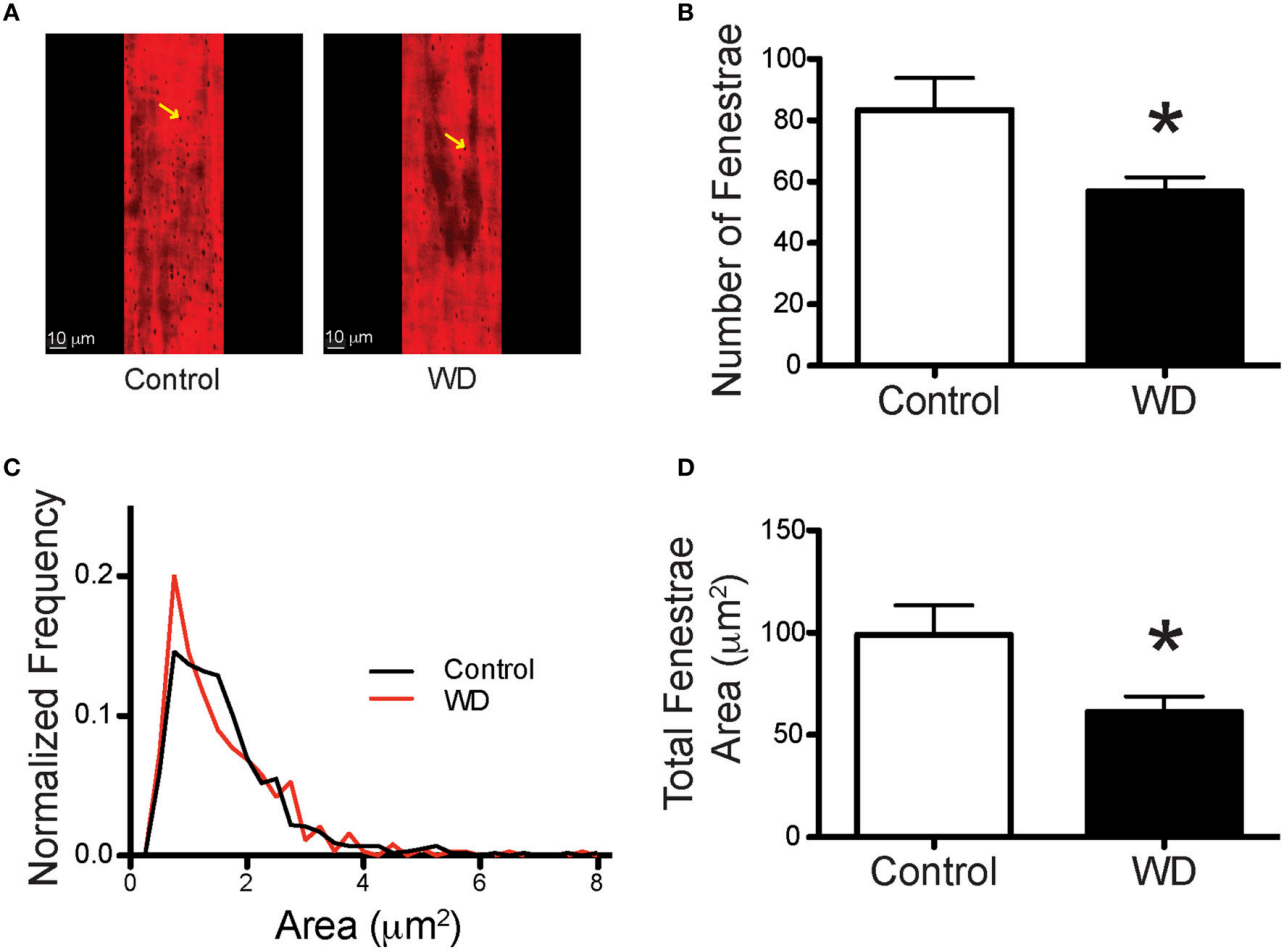
**WD-feeding decreased the number and area of fenestrae within the IEL of mesenteric arteries. (A)** Representative images of the IEL stained with Alexa633 that show the presence of fenestrae (yellow arrows) in the wall of mesenteric arteries from a ND- (Control) and a WD-fed mouse. **(B)** Comparison of the mean number of fenestrae per unit area in the IEL of mesenteric arteries isolated from ND- (Control) and WD-fed mice. **(C)** Frequency distribution of the number of fenestrae by area, binned in 1 μm^2^ increments and contained within the IEL of mesenteric arteries from ND- (Control) and WD-fed mice. **(D)** Comparison of the mean total fenestrae area per IEL unit area in mesenteric arteries from ND- (Control) and WD-fed mice. In B and D, data are expressed as means ± SEM of *n* = 9 ND- (Control) and *n* = 10 WD-fed mice. ^*^P < 0.05 vs. Control.

To determine the potential contribution of cells and specific ECM components on the mechanical changes associated with WD-feeding on mesenteric resistance arteries, we imaged (Figures 4A–D) and quantified VSMC nuclei, VSMC F-actin content, elastin and collagen in arteries isolated from WD- and ND-fed mice. There were no significant differences in the number of VSMCs, F-actin content or VSMC F-actin to nuclei ratios in the medial layer of arteries from WD- vs. ND-fed mice (Figures 4E–G). However, the volume occupied by elastin-associated fluorescence was greater (*P* < 0.05) in vessels from WD- vs. that in ND-fed mice, indicating an increase in elastin volume for the WD cohort (Figure 4H). Collagen content did not differ between the groups (Figure 4I), but elastin to collagen ratios were significantly greater in the WD cohort (Figure 4J). Moreover, the number of fenestrae present in the IEL of arteries from mice fed a WD was significantly (*P* < 0.05) fewer than that in vessels from the ND-fed mice (Figures 5A,B). There was also a shift in the distribution of size toward smaller (~1 μm^2^) fenestrae in vessels from the WD-fed mice (Figure 5C). Consequently, the fenestrae total area was significantly smaller for the WD compared to the ND group (Figure 5D).

### WD-feeding increased plasma neuraminidase and mesenteric artery TGF-β content

**Figure 6:**
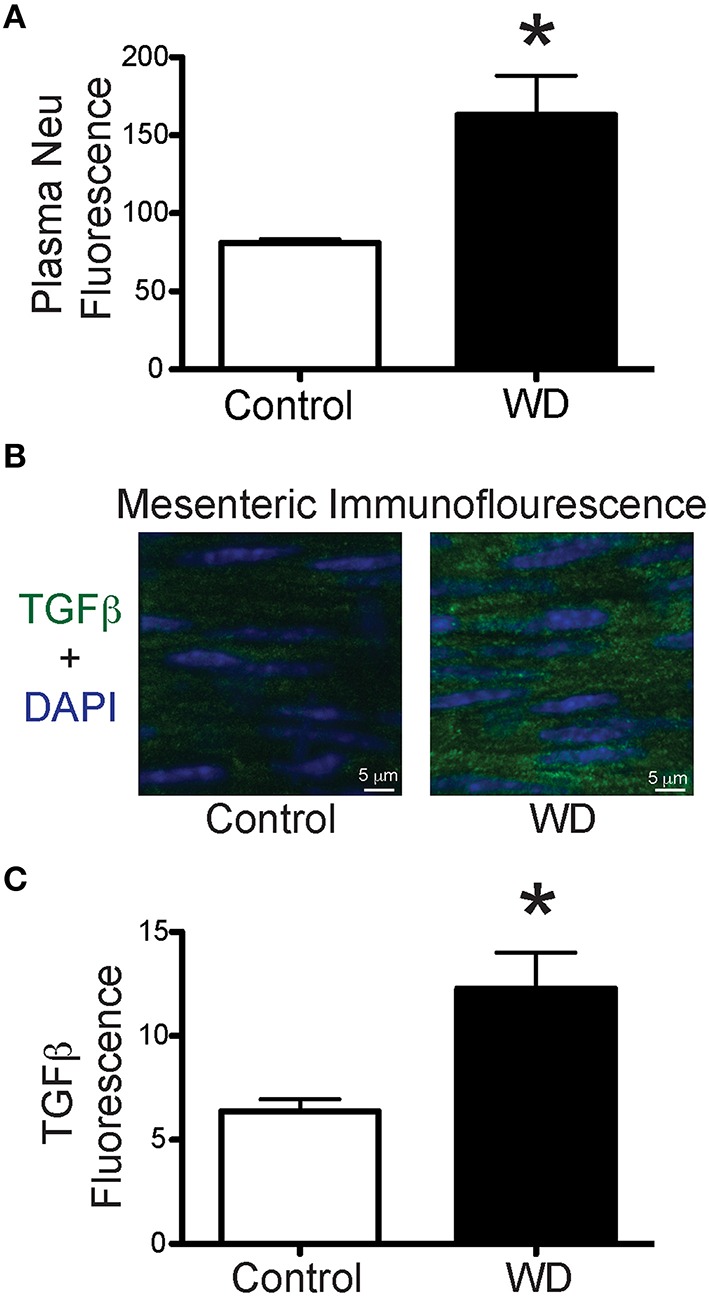
**WD-feeding increased plasma neuraminidase activity and presence of TGF-β in mesenteric arteries. (A)** Level (arbitrary units) of fluorescence induced by neuraminidase (Neu) activity in plasma obtained from ND- (Control, *n* = 4) and WD-fed (*n* = 4) mice. **(B)** Representative images of immunofluorescence associated with the presence of TGF-β (green) and nuclei (DAPI, blue) in mesenteric arteries from a ND- (Control) and a WD-fed mouse. **(C)** Comparison of the mean TGF-β-associated fluorescence in mesenteric arteries from ND- (Control, *n* = 7) and WD-fed (*n* = 7) mice. In A and C, data are expressed as means ± SEM. ^*^P < 0.05 vs. Control.

In order to determine if consumption of a WD had an effect on the circulation levels of the enzyme associated with the elastin receptor complex, we measured neuraminidase levels in plasma obtained from WD- and ND-fed mice. Plasma from the WD group had a greater (*P* < 0.05) level of neuraminidase activity than that in the ND cohort (Figure 6A). In order to identify potential mediators of elastogenesis downstream of neuraminidase activation, we also measured the amount of TGF-β present within the wall of mesenteric arteries and found that vessels from WD-fed mice had greater (*P* < 0.05) TGF-β content than those from ND-fed mice (Figures 6B,C).

### Neuraminidase increases tropoelastin and TGF-β production in isolated VSMCs

**Figure 7:**
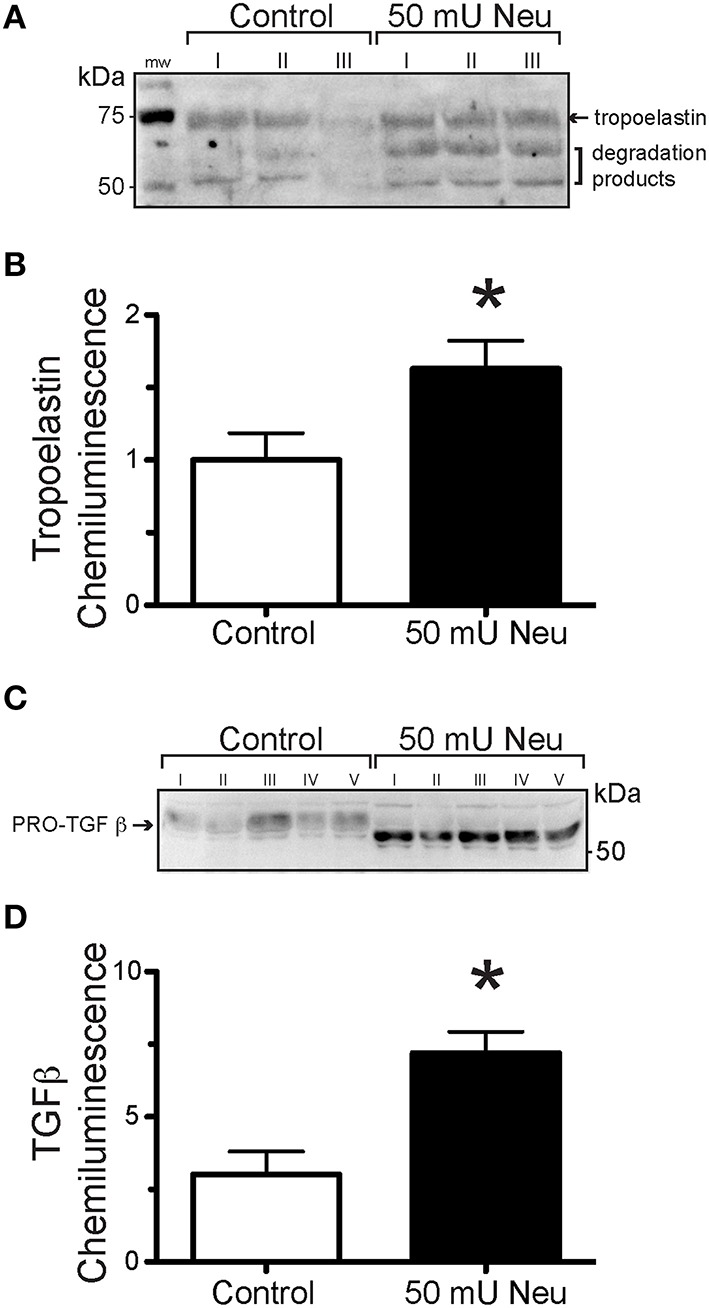
**Neuraminidase-exposure increased elastogenesis and TGF-β production in VSMCs in culture. (A)** Representative image of a Western Blot using the extract of VSMCs not exposed (Control) or exposed to 50 mU neuraminidase (Neu) and probed with anti-tropoelastin antibody. **(B)** Densitometric quantification of tropoelastin content in Western Blots using the extract of VSMCs not exposed (Control, *n* = 6) or exposed to 50 mU neuraminidase (Neu, *n* = 6). Data are expressed as means, normalized to control, ± SEM of the sum chemiluminescent intensity of all three protein bands. **(C)** Representative image of a Western Blot using the extract of VSMCs not exposed (Control) or exposed to 50 mU neuraminidase (Neu) and probed with anti- TGF-β antibody. **(D)** Densitometric quantification of TGF-β content in Western Blots using the extract of VSMCs not exposed (Control, *n* = 5) or exposed to 50 mU neuraminidase (Neu, *n* = 5). Data are expressed as means ± SEM. ^*^P < 0.05 vs. Control.

In order to determine the effect of neuraminidase on the production of tropoelastin and TGF-β by VSMCs, we exposed VSMC in culture to neuraminidase (50 mU) and measured the content of tropoelastin and TGF-β in the culture media. VSMCs exposed to neuraminidase produced greater amounts of tropoelastin and TGF-β than those exposed to vehicle control (Figures 7A–D). The TGF-β present in the culture media of VSMCs exposed to neuramindase had a gel mobility shift toward a lower molecular weight, indicative of deglycosylation, presumably due to the sialidase activity of the exogenously applied neuraminidase (Carlson et al., 2010).

## Discussion

In the present study, a highly translational WD (high in fat and sugar) was used to promote over-nutrition in mice and to determine its effects on resistance artery mechanical properties and structure. Our primary finding was that male mice fed a WD for 16 weeks had stiffer mesenteric arteries than mice fed a ND. This stiffening of the mesenteric arteries was associated with a reduction in the number and total area of fenestrae within their IEL, as well as an increased amount of elastin present in the vascular wall. Surprisingly the amount of collagen was not changed, nor was the number of VSMCs or F-actin content within the medial layer of the vessels. A previous study examining structural changes in mesenteric arteries in a different model of obesity that is associated with diabetes (db/db mice) also found a significantly increased arterial stiffness at high pressure (125 mmHg) in the obese cohort (Souza-Smith et al., 2011). In that study the mesenteric arteries from the obese cohort displayed an increased passive luminal diameter with an increased wall cross-sectional area (outward hypertrophic remodeling) and a decreased wall to lumen ratio without changes in collagen content. Elastin content was not measured. In our present study, we did not observe outward remodeling, but we did see a trend for a reduction in the wall to lumen ratio for the WD cohort that was associated with a significant reduction in wall cross-sectional area and no changes in collagen content. This suggests that similar structural alterations are present in the mesenteric vasculature of hyperphagic db/db mice and mice fed a WD in that there is a stiffening of the vascular wall at high pressures and an overall reduction in the amount of wall material normalized to luminal diameter without changes in collagen content. We did not find any functional changes in mesenteric vessels from WD- vs. ND-fed mice as neither their constriction (KCl, phenylephrine) or relaxation (insulin, SNP) responses were significantly different. This suggests that, in animals fed a WD, structural alterations to the IEL and vascular stiffness in mesenteric arteries precede functional changes to vasoactive stimuli. However, we cannot rule out that all endothelial dependent dilation pathways are unaffected by a 16 week WD time course. Future WD studies that test both additional vasoconstriction and vasodilation pathways and extend the duration of WD treatment need to be performed to confirm this assumption.

We have previously reported that male mice fed the same WD as in the present study had different remodeling responses in femoral vs. coronary arteries. WD-feeding was associated with stiffening and a reduced elastin to collagen ratio in femoral but not in coronary arteries. The only common WD-induced changes on the mechanical and structural characteristics in the femoral arteries reported by Bender et al. (Bender et al., 2015) and the mesenteric arteries in our present study are an increased elastic modulus (stiffening) and a reduction in the number and area occupied by fenestrae in the IEL. A reduction in the number and area of fenestrae in the IEL of vessels from hypertensive rodents has also been reported (Briones et al., 2009). The mechanism(s) leading to decrease fenestrae numbers, as well as the reduction in total fenestrae area, is/are yet to be determined. In this study, the changes in the fenestration of the mesenteric vasculature coincided with an increase in elastin volume. A similar link between decreased fenestra area and increased elastin content has been reported in two separate rat models of hypertension. Neonatal spontaneously hypertensive rats (SHRs) were found to have increased elastin-derived fluorescence in carotid arteries and this was associated with a decrease in the size of individual fenestrae (Arribas et al., 2008). A 2-week infusion of angiotensin II to induce hypertension in Wistar rats also resulted in a reduced number of fenestrae and a reduction in the total fenestrae area in the mesenteric vasculature and this coincided with an increase in the relative area occupied by elastin (Briones et al., 2009). The current observation of increased deposition and/or reorganization of elastin within the IEL and a decrease in the number of fenestrae suggests a mechanism in which elastin deposition is targeted to sites of fenestration. It has previously been shown that in the developing mouse aorta, newly synthesized elastin preferentially accumulates at extant fenestrae (Davis, 1995). Though, how this occurs is unclear. Conceivably the borders of fenestrae are biochemically favorable for the polymerization/crosslinking of newly synthesized tropoelastin monomers. Alternatively, the molecules or enzymes that facilitate polymerization of tropoelastin monomers, such as fibulins and lysyl oxidase (Horiguchi et al., 2009) and/or transglutaminase 2 (Kielty, 2006), could preferentially be targeted to sites of fenestration. Additional studies are necessary to define how and where newly synthesized elastin is deposited in the microvasculature.

Regardless of the underlying mechanism of deposition at sites of fenestration, depending upon the amount of elastin deposited, one would expect the size of the fenestrae to be reduced as well as eventually the overall number of fenestrae. In our previous study we found that fenestrae in the femoral arteries from WD-fed mice were smaller than those in the ND-fed mice. In the present study we did not find a significant difference in the mean size of fenestrae, but the frequency distribution of the individual fenestrae was shifted so that an increased frequency of smaller fenestrae approximately 1 μm^2^ in area occurred in the WD-fed cohort compared to the ND-fed cohort. Interestingly, differences in the amount of signal for production of elastin in response to diverse dietary interventions to induce obesity varies between vascular beds. In our previous study we did no see an increase in overall elastin content in femoral arteries. Others found that feeding a high fat diet to mice for 8 weeks did not alter the elastin content of mesenteric arteries (Dantas et al., 2014). In comparison, feeding a high fat diet to pigs for 6 months was associated with a significant elevation of mRNA for elastin in coronary resistance microvessels (Trask et al., 2012). A similar increase in elastin mRNA in these vessels was found in db/db mice (Katz et al., 2011). This suggests that elastin production and accumulation in blood vessels in obesity varies according to the characteristics of the obesity-inducing stimulus, the time of stimulation and the vascular bed analyzed, with an apparent preference for greater elastin deposition in resistance vessels.

Additional studies emphasize a differential effect of obesity or diet on the mechanical and structural characteristics of conduit vs. resistance arteries and on vessels from different vascular beds. For example, thoracodorsal arteries from 8 to 10 week old male mice, fed a high fat diet for 6 weeks, displayed reduced compliance (increased stiffness) in contrast to larger, carotid arteries from the same animals in which compliance was unaffected by the high fat diet treatment (Billaud et al., 2012). In a study by Lesniewski et al., carotid arteries from 5 month-old, male mice fed a WD had an increased incremental stiffness compared to carotid arteries from mice fed control chow. In addition, they report that the carotid arteries from the WD-fed cohort also had impaired responses to acetylcholine (Lesniewski et al., 2013). Though acetylcholine responses were not tested in our present study, we have observed that mesenteric arteries from female mice, fed a WD for 16 weeks, are more responsive to acetylcholine than mesenterics from control-fed females (unpublished data). In addition, we report here that insulin responses were not blunted in mesenteric arteries from WD-fed males (Figure 2C), suggesting that endothelium-dependent vasodilatory pathways were not affected by WD under our experimental conditions. Because many parameters vary in the experimental procedures used in different studies, it is difficult to assess the mechanisms that may account for the differential effects that diet-induced obesity may have on vascular function and mechanics. Some of the factors that should be considered include the characteristics of the diet, the sex of animals used, the age of the animals at the time of diet initiation, the duration of the diet and the effects that diet may have on the hemodynamics of the vascular bed studied. For example, it has been shown that blood flow is increased in the mesenteric circulation of hyperphagic obese mice (Souza-Smith et al., 2011). Therefore, it is possible that changes in hemodynamics caused by WD-feeding in the mesenteric circulation may be a factor that affects the progression of remodeling in this vascular bed, but this needs to be experimentally determined.

Of significant interest are the mechanisms that initiate remodeling of the ECM within the microvasculature, specifically what changes the equilibrium of elastin synthesis/degradation to favor increased elastin deposition and IEL remodeling in WD-induced obesity? Elastin synthesis is regulated at multiple levels including: DNA transcription, mechanisms that affect the stability of existing elastin mRNA, mechanisms that affect the enzymes that mediate tropoelastin monomer polymerization and also via mechanisms that regulate ECM proteases that actively degrade elastin (Kielty et al., 2002). In this regard, it has been demonstrated that mice fed a high fat diet have increased levels of elastin peptides in the circulation (Blaise et al., 2013). A subset of these fragments are biologically active and have been implicated in the progression of a number of vascular diseases including atherosclerosis (Baydanoff et al., 1987) and diabetes (Nicoloff et al., 2007). It is hypothesized that these peptides exert their activity by binding to the elastin receptor complex, a heterotrimeric plasma membrane receptor, comprised of the elastin-binding protein, protective protein/cathepsin A and neuraminidase. Once activated, the receptor modulates a number of downstream processes, including the positive regulation of elastogenesis, via the sialidase activity of neuraminidase (Hinek et al., 2006). Based on this background information and our interest in identifying potential mediators of elastogenesis, we measured the circulating levels of neuraminidase and found that plasma from the WD-fed cohort had a significantly higher sialidase activity compared to plasma from the ND-fed group. This suggests that activation of the elastin receptor complex may be increased in WD-induced obesity.

Increased neuraminidase activity is associated with increased production and activation of TGF-β. In addition, neuraminidase and TGF-β have been implicated in elastogenesis. TGF-β in particular stabilizes elastin mRNA in rat (McGowan et al., 1997) and human lung fibroblasts (Kucich et al., 1997) via a pathway that includes Smads, protein kinase C, and p38 (Kucich et al., 2002). ECM enzymes required for polymerization of tropoelastin monomers are transcriptionally up regulated (Kuang et al., 2006) and secreted (Boak et al., 1994) in response to TGF-β. It has also been shown that TGF-β suppresses elastin degradation via inhibition of matrix metalloproteinase-9 (Alvira et al., 2011). Furthermore, we have previously shown that TGF-β protein is increased in both cardiac tissues and in femoral arteries of WD- compared with ND-fed mice (Bender et al., 2015; Jia et al., 2015). Consequently, we measured TGF-β content in the mesenteric arteries we studied here and found that vessels from WD-fed mice had greater amounts of this cytokine in their walls compared with those from ND-fed mice.

In order to determine if our observations that WD-feeding increased plasma neuraminidase and arterial content of TGF-β and elastin implicates a causative relationship, we tested the effects of exposing VSMCs to neuraminidase on their production of TGF-β and tropoelastin. We found that the addition of neuraminidase to cultured VSMCs increased the secretion of TGF-β and tropoelastin into the culture media. This is consistent with a report from Hinek et al. indicating that exogenously added neuraminidase significantly increased elastin fiber deposition in cultured aortic smooth muscle cells (Hinek et al., 2006, 2008), and with results that indicate neuraminidase increases TGF-β production and activation (Carlson et al., 2010). Moreover, these results support a model in which WD-induced obesity upregulates elastases, which generate elastin fragments that activate the elastin receptor complex. This in turn leads to an elevation in circulating levels of neuraminidase, which increases vascular TGF-β production/activity, thereby upregulating elastogenesis and thus mediating remodeling of the mesenteric resistance vasculature.

The physiological significance of reduced total fenestrae area is poorly understood. One investigative group reported that conduit/carotid arteries from 1-week old SHRs had decreased fenestration and this was coincident with increased arterial stiffness compared with age matched control rats (Arribas et al., 2008). Moreover, these ECM differences were manifest prior to any significant changes in blood pressure between the controls and SHRs. Similarly, in a study by Weisbrod et al., the authors also report that arterial stiffening increased prior to the onset of hypertension in mice fed a WD (Weisbrod et al., 2013). Using radio telemetry, we previously demonstrated that WD-feeding induced a modest increase (8.2%) in systolic blood pressure, in a different cohort of mice at the same age and undergoing the same treatment protocol (Nistala et al., 2014). In humans, a classification of hypertension entails an approximate 16% increase in systolic blood pressure (120 mmHg for normotensive vs. 140 mmHg for hypertensive). Thus, we infer that the mice in this study were in the process of developing hypertension. In contrast, the observed ECM remodeling was well-progressed, as the difference in fenestration was substantial between the two treatment groups, and likely was the result of incremental increases in elastin deposition over time. Therefore, our findings suggest that reorganization of the ECM may be one of the first pathological events that triggers stiffening and the subsequent development of hypertension in obesity.

We currently do not know whether the reduced fenestration of the IEL in mesenteric arteries mediates the increased vascular stiffness we observed. We are intrigued by the possibility that impaired signaling between the intima and medial layers may play a role. Fenestrae allow for the exchange of macromolecules between endothelial cells and the VSMCs that comprise the media. They also allow for the projection of endothelial cell membranes to make contact with VSMC membranes. It is posited that these myo-endothelial interfaces allow for the transmission of endothelium-derived factors across the IEL (Sandow et al., 2002; Straub et al., 2012). A reduction in total fenestra area would likely affect the time it takes for diffusible substances to transit between the intima and medial layers, and decreases in the number of fenestrae would decrease the number of contacts for the transmission of vasoactive factors. Thus, a plausible explanation for how a decrease in total fenestrae area could trigger arterial stiffening may be via attenuation in the diffusion of vasoactive factors and myo-endothelial communications. However, this hypothesis would require extensive testing, as our observations indicate there were no impairments in the endothelium-dependent vasodilatory responses to insulin in WD-fed mice. Alternatively, it has recently been postulated that arterial stiffness may arise, in part, from changes in the actin cytoskeleton that affect the intrinsic mechanical properties of individual VSMCs (Sehgel et al., 2013). Although we did not observe any significant changes in the amount of F-actin present in the medial layer of arteries from mice fed a WD, it is possible that the arrangement of cortical cytoskeletal structure may have changed and made VSMCs stiffer.

It is also plausible that the observed increase in stiffness is not directly related to changes in the degree of IEL fenestration. The pro-fibrotic activity of TGF-β could lead to increased cross-linking of ECM proteins, and thereby contribute to vascular stiffness. In support of this alternative hypothesis, the addition of TGF-β to human cell cultures has been shown to significantly increase the secretion of the ECM cross linking enzyme, transglutaminase II (George et al., 1990; Jung et al., 2007). A report from Weisbrot et al. demonstrated that transglutaminase activity was increased in aortic extracts from mice fed a comparable WD and of a similar age to the mice in this study, and this was coincident with both increased aortic ECM cross links and aortic stiffness (Weisbrod et al., 2013). We have previously shown that transglutaminase activation increases resistance artery stiffness in rat cremasteric vessels (Castorena-Gonzalez et al., 2014). However, whether crosslinking of the ECM and/or reduced communication between the endothelium and media layers play a role in the increased stiffness observed in WD fed mice, requires further investigation and is beyond the scope of this study.

## Study limitations

The following limitations of this study should be considered. Crosslinking of the ECM was not measured. Increases in cross-linking would presumably affect vessel stiffness. We also did not perform cellular stiffness assays to determine if a change in the stiffness of the cells contained within the wall of the vessel contribute to the increased incremental modulus of elasticity that we reported. Lastly, we measured collagen using a multi-photon laser to generate a second harmonic signal. This technique is limited to quantifying fibrillar collagen and is not a measure of the total collagen content within the ECM, that would include collagen fibers that do not harmonize. In addition to elastin and collagen, other components of the ECM may also contribute to the overall stiffness of the vessel and these as well were not assessed.

## Conclusion

In this study, we demonstrate that a WD rich in fat and sugars induces remodeling of the ECM in mesenteric resistance arteries, via an increase in elastin volume and a concomitant decrease in fenestrations within the IEL. These changes are associated with stiffening of the vascular wall in absentia of functional changes to vasoactive stimuli. In addition we report that a WD increases plasma neuraminidase levels and that neuraminidase promotes the production of tropoelastin and TGF-β by VSMCs. We hypothesize that the chronic release of pro-inflammatory cytokines, as a consequence of the obese state, coupled with elevated serum sialidase activity initiates remodeling of the ECM within the vasculature, which lead to alterations in vascular stiffness and the subsequent development of adverse cardiovascular pathologies.

## Author contributions

LM and JS conceived and designed the study, analyzed and interpreted data and edited the manuscript. CF, JC, and FR performed experiments made figures and wrote the manuscript. GJ and MH helped design and performed animal and molecular experiments and edited the manuscript. CR designed and performed image analyses and edited the manuscript. All authors approved the final version of the manuscript.

### Conflict of interest statement

The authors declare that the research was conducted in the absence of any commercial or financial relationships that could be construed as a potential conflict of interest.
